# Clinical Features of Central Nervous System Complications Caused by the SARS‐CoV‐2 Omicron Variant

**DOI:** 10.1002/iid3.70247

**Published:** 2025-08-13

**Authors:** Tingting Li, Zhirong Deng, Qinfu Zhang, Xiaoying Qi, Wei Deng, Zunge Wu, Chuli Xiao, Weiqiang Zheng, Chuanghong Ke, Huanqin Han

**Affiliations:** ^1^ Department of Infectious Diseases Affiliated Hospital of Guangdong Medical University Zhanjiang China; ^2^ Department of Infectious Disease People's Hospital of Longhua Shenzhen China; ^3^ Children's Medical Center Affiliated Hospital of Guangdong Medical University Zhanjiang China

**Keywords:** adult, cerebrospinal fluid, clinical features, CNS complications, omicron variant

## Abstract

**Background:**

This study investigated clinical characteristics and cerebrospinal fluid (CSF) features of central nervous system (CNS) complications in adults caused by the severe acute respiratory syndrome coronavirus 2 (SARS‐CoV‐2) Omicron variant.

**Methods:**

We retrospectively examined adult patients with coronavirus disease‐2019 (COVID‐19), with or without CNS complications, hospitalized in China from December 2022 to August 2023.

**Results:**

Twenty‐five patients with CNS complications (9 severe, 16 non‐severe) and 50 patients without CNS complications were examined. The percentage of patients with severe/critical COVID‐19 was relatively higher in the CNS complications group (24% [6/25] vs. 12% [6/50]). Procalcitonin and creatine kinase levels, and leukocyte and neutrophil counts were significantly higher in patients with CNS complications. Having severe CNS complications was associated with older age, comorbidities, and elevated creatine kinase and d‐dimer levels. Among 22 patients with CNS complications who underwent CSF testing, 10 (45%) had abnormal CSF cytology or biochemistry—including 9 (41%) with elevated protein levels—and 2 (9%) had increased white blood cell counts. Among the CSF samples tested, 2 (12%) and 3 (18%) tested positive for SARS‐CoV‐2 and autoantibodies respectively.

**Conclusions:**

Older age, comorbidities, and elevated serum creatine kinase and d‐dimer levels were risk factors for severe CNS complications in adult patients with COVID‐19. SARS‐CoV‐2 was not detected in the CSF of most patients with CNS complications.

## 1 Introduction

Toward the end of 2021, the severe acute respiratory syndrome coronavirus 2 (SARS‐CoV‐2) Omicron variant emerged and rapidly spread globally. The Omicron variant is distinguished by its heightened transmissibility and diminished pathogenicity. Based on statistical data provided by the Chinese Center for Disease Control and Prevention [1] from September 2022 to June 2023, SARS‐CoV‐2 occurrence within mainland China was exclusively associated with the Omicron variant.

Several studies have reported that SARS‐CoV‐2 infection has the potential to induce a cytokine storm, thereby engendering a cascade of physiological events culminating in multiorgan dysfunction and potentially leading to organ failure. SARS‐CoV‐2 encompasses a wide range of conditions, including acute respiratory distress syndrome, cardiovascular disorders, immune dysregulation, and neurological impairment [2, 3, 4] A growing body of evidence has shown that SARS‐CoV‐2 has the capacity to elicit neurological manifestations, with a high proportion of patients showing clinical features such as cephalalgia, vertigo, myalgia, altered cognitive states, and aberrations in psychological functioning [5, 6, 7] In late 2019, in Wuhan, China, Mao et al. [8] reported that 36.4% of patients with what later became known as coronavirus disease‐2019 (COVID‐19) had neurological complications. Likewise, during the same timeframe, Yan et al. [9] reported that 30.3% of patients with nonsevere infections at the Wuhan Fangcang Temporary Hospital had neurological manifestations. However, during a period of heightened prevalence of the Omicron variant, Shen et al. [10] reported a notably elevated proportion (48.1%) of neurological manifestations among individuals with mild‐to‐moderate COVID‐19 in Shanghai, China. This observation suggests that neurological complications are more common in individuals infected with the Omicron variant than in those infected with the earlier SARS‐CoV‐2 strains and variants. However, information on post‐neurological symptoms associated with the Omicron strain remains limited.

The SARS‐CoV‐2 Omicron variant has neuroinvasive, neurotropic, and neurotoxic potential and can cause severe central nervous system (CNS) complications such as encephalopathy, encephalitis, and autoimmune neurological diseases. Kim et al. [11] revealed that during the Omicron variant surge, encephalopathy (68.5%) was the most common neurological complication observed in post‐COVID‐19 patients in New York City. A multicenter study [12] has also reported that acute encephalopathy is the most common neurological disorder during both the prodromal and acute respiratory phases of the Omicron variant infection. This indicates a high incidence of CNS complications in the Omicron epidemic. However, there is a paucity of studies elucidating the clinical manifestations and treatment outcomes of CNS complications triggered by adult‐onset SARS‐CoV‐2 Omicron variant infections.

Therefore, this study investigated the clinical manifestations of CNS complications in adults with COVID‐19 caused by SARS‐CoV‐2 Omicron variant infection. The study also investigated the patients' cerebrospinal fluid (CSF) and metagenomic next‐generation sequencing (mNGS) results to provide comprehensive diagnostic insights.

## 2 Methods

### 2.1 Patients and Groups

This retrospective study included patients with COVID‐19 complicated by CNS complications admitted to a university hospital in China between December 2022 and August 2023. The patients were classified into a CNS complications group and non‐CNS complications control group. The control group consisted of patients hospitalized with COVID‐19 without CNS complications during the same period. The control group was selected using stratified random sampling. The patients were confirmed by the Chinese Center for Disease Control and Prevention to be infected with the Omicron BA.2.5 or XBB variants.

Subgroups were created based on the Glasgow Coma Scale (GCS) score, with patients with a GCS score ≤ 8 being classified as having severe CNS complications (severe group), and patients with a GCS score > 8 being classified as having mild‐to‐moderate CNS complications (non‐severe group). The GCS score involves three different tests: eye opening, verbal responses, and motor responses [13]

The study was approved by the Ethics Committee of Guangdong Medical University (Approval No.: PJKT2024‐033) and was carried out in accordance with the guidelines of the Declaration of Helsinki. Informed consent was obtained from all participants.

### 2.2 Inclusion and Exclusion Criteria

Patients were required to fulfill the following criteria simultaneously: (1) positive SARS‐CoV‐2 reverse transcription‐polymerase chain reaction or antigen test results from pharyngeal swabs, confirming COVID‐19 diagnosis; (2) age > 18 years; and (3) manifestations of COVID‐19‐associated cognitive decline, change in consciousness level, behavioral abnormalities, seizures, psychiatric disorders, or decreased muscle strength within 4 weeks following the onset of COVID‐19. Patients with cerebrovascular accidents or peripheral neuropathy, such as Guillain–Barré syndrome, were excluded.

The severity of COVID‐19 was based on the COVID‐19 Severity Classification [14]: (1) Mild – upper respiratory tract infection without pneumonia; (2) moderate—persistent high fever ( > 3 days) and/or respiratory symptoms with respiratory rate < 30 breaths/min, SpO_2_ > 93% on room air, and characteristic pneumonia findings on imaging; (3) severe—dyspnea with respiratory rate ≥ 30 breaths/min, SpO_2_ ≤ 93% on room air, PaO_2_/FiO_2_ ≤ 300 mmHg, or > 50% radiographic progression within 24–48 h; (4) critical—respiratory failure requiring mechanical ventilation, shock, or multiorgan failure necessitating intensive care.

### 2.3 Data Collection

Patient data were collected on epidemiological characteristics, medical history, duration of hospitalization, laboratory test results, and chest imaging findings. The laboratory data included levels of serum procalcitonin (PCT), C‐reactive protein (CRP), creatine kinase (CK), creatinine, bilirubin, fibrinogen, and plasma d‐dimer; hematology (leukocyte, neutrophil, lymphocyte, monocyte, and red blood cell counts, and hemoglobin level), prothrombin time, and partial thromboplastin time. The CSF results, including total protein level and CSF leukocyte count, were collected for patients who underwent CSF testing.

### 2.4 Statistical Analysis

Data normality was assessed using Shapiro–Wilk tests and Q‐Q plots. Continuous variables are reported as mean ± standard deviation if they followed a normal distribution, or median and interquartile range (IQR) if they were non‐normally distributed. Independent samples *t*‐tests or Mann–Whitney U‐tests were used to assess the significance of observed differences between groups. Categorical variables are expressed as percentages, and groups were compared using χ^2^ tests. The threshold for statistical significance was set as a two‐sided *p* < 0.05. Data analysis was performed using SPSS version 26.0 (IBM Corp., Armonk, NY, USA).

## 3 Results

### 3.1 Demographic and Clinical Characteristics

This study included 75 patients diagnosed with SARS‐CoV‐2 Omicron infection. The CNS complications group consisted of 25 patients, including 14 men and 11 women, with a mean age of (47.3 ± 17.1) years. The non‐CNS complications group consisted of 50 patients, including 28 men and 22 women, with a mean age of (50.7 ± 17.3) years. The age, sex, booster immunization (having received the third dose of the vaccine), and co‐infection with bacteria distributions of the two groups showed no significant differences.

In the CNS complications group, 6 patients (24%) had severe/critical COVID‐19, and 19 (76%) had mild/moderate COVID‐19. Evidence of viral pneumonia was identified on imaging in 80% of patients. In the non‐CNS complications group, 6 patients (12%) had severe/critical COVID‐19, and 44 (88%) had mild/moderate COVID‐19. The incidence rate of viral pneumonia was 78.6%. The proportions of patients with mild, moderate, and severe/critical COVID‐19, and with viral pneumonia did not differ significantly between the two groups (Table 1).

**Table 1:** Laboratory and radiological findings in patients with COVID‐19 with and without associated CNS complications.

	Normal range	Without CNS complications (*n* = 50)	With CNS complications (*n* = 25)	*p* value
Age, years	—	50.72 ± 17.31	47.32 ± 17.10	0.423
Sex, (*n*, %)	—			> 0.999
Male		28 (56%)	14 (56%)	
Female		22 (44%)	11 (44%)	
Typing, (*n*, %)	—			0.316
Proportion of severe or critical		6 (12%)	6 (24%)	
Proportion of mild or moderate		44 (88%)	19 (76%)	
Booster immunization, (*n*,%)	—	26 (52%)	17 (68%)	0.222
Symptom onset‐to‐admission interval (days)		7 (4–9)	5 (3–11)	0.608
Viral pneumonia on chest CT or radiography	—	33/42 (78.6%)	20/25 (80%)	> 0.999
Co‐infection with bacteria		7 (14.0%)	4 (16.0%)	> 0.999
Fever	—	22 (44%)	7 (28%)	0.180
Dizziness	—	7 (14%)	3 (12%)	> 0.999
Headache	—	9 (18%)	5 (20%)	> 0.999
Procalcitonin, ng/mL	< 0.05	0.05 (0.03–0.10)	0.13 (0.05–0.40)	0.032*
C‐reactive protein, mg/L	0–4	22.83 (11.78–102.99)	54.02 (7.03–112.11)	0.521
WBC count, ×10^9^/L	4–10	6.23 ± 2.24	9.41 ± 4.54	< 0.001***
Neutrophil granulocyte count, ×10^9^/L	2–7	3.76 (2.70–5.43)	6.23 (4.00–9.83)	0.001**
Lymphocyte count, ×10^9^/L	0.8–4.0	1.27 ± 0.65	1.53 ± 0.90	0.208
Platelet count, ×10^9^/L	100–300	226.5 (175.5–271.3)	246.0 (161.5–307.5)	0.629
Creatine kinase, U/L	25–170	62.9 (40.8–107.25)	234.0 (77.5–1075.1)	< 0.001***
Total protein, g/L	60–82	67.6 ± 6.6	64.9 ± 6.8	0.107
Albumin, g/L	38–54	39.3 ± 5.5	37.3 ± 6.0	0.284
Globulin, g/L	20–40	27.9 (25.2–30.2)	26.8 (23.5–30.6)	0.402
Prothrombin time, s	10.6–14.3	12.8 (12.4–13.5)	13.2 (12.5–14.5)	0.072
Fibrinogen, g/L	2–4	4.6 ± 1.5	3.9 ± 1.4	0.062
Activated partial thromboplastin time, s	26–40	35.5 (33.0–37.7)	35.2 (32.2–38.1)	0753
Thrombin time, s	14–21	18.0 (17.0–18.8)	17.2 (16.1–18.9)	0.214
d‐dimer, μg/mL	< 0.02	0.6 (0.3–1.0)	0.6 (0.4–2.6)	0.151
Fibrin degradation products, μg/mL	0–5	2.5 (1.9–4.0)	2.0 (1.5–3.6)	0.374
Hospital days, median (IQR), days		6.5 (4–9)	10 (8–17)	0.001**

*Note:* Data are presented as median (interquartile range), mean ± standard deviation, or *n* (%).

Abbreviations: CNS, central nervous system; CT, computed tomography; IQR, interquartile range; WBC, white blood cell.

* *p* < 0.05

** *p* < 0.01

*** *p* < 0.001.

These results suggested that the general characteristics and severity of COVID‐19 were similar between the two groups. However, the CNS complications group had a significantly longer hospital stay than the non‐CNS complications group (median: 10 days vs. 6.5 days; *p* < 0.001).

### 3.2 Symptoms and Laboratory Analysis

Patients with and without CNS complications had similar incidences of viral pneumonia (80% vs. 78.6%), fever (28.0% vs. 44.0%), dizziness (12.0% vs. 14.0%), and headache (20.0% vs. 18.0%) (Table 1). Within the CNS complications group, seizures demonstrated the highest incidence (24%), followed by syncope with an incidence rate of 12%.

The PCT levels were significantly higher in patients in theCNS complications group than in those in the non‐CNS complications group (*p* = 0.032), whereas the peripheral blood leukocyte, neutrophil, and monocyte counts and serum CK levels were significantly higher among patients in the CNS complications group than among those in the non‐CNS complications group (Table 1).

Of the 25 patients in the CNS complications group, 9 had severe CNS complications, and 16 had non‐severe CNS complications. Patients in the severe group were significantly older than those in the non‐severe group (*p* < 0.001), and the proportion of patients in the severe group with two or more chronic underlying diseases was significantly higher than that in the non‐severe group (44% vs. 6%; *p* = 0.04; Table 2).

**Table 2:** Demographic and clinical characteristics in patients with CNS complications according to the severity of CNS complications.

Characteristic	Severe (*n* = 9)	Non‐severe (*n* = 16)	*p* value
Age (years), mean (SD)	61.2 ± 9.9	39.5 ± 15.3	< 0.001***
Sex, *n* (%)			0.115
Female	6 (67%)	5 (31%)	
Male	3 (33%)	11 (69%)	
≥ 2 comorbidities, *n* (%)^a^	4 (44%)	1 (6%)	0.04*
Co‐infection with bacteria	2 (22.2%)	2 (12.5%)	0.602
Booster immunization, (*n*,%)	4 (44.4%)	13 (81.3%)	0.087
Hospital days, median (IQR)	10 (8–20)	9.5 (7.3–13.8)	0.493

Abbreviations: CNS, central nervous system; IQR, interquartile range; SD, standard deviation.

^a^ Comorbidities included hypertension, diabetes, neurological disorders, chronic liver disease, and chronic lung disease.

Regarding the laboratory test results, patients in the severe group had significantly higher aspartate aminotransferase, CK, and d‐dimer concentrations than those in the non‐severe group (Table 3).

**Table 3:** Laboratory test results and chest radiography findings in patients with CNS complications and SARS‐CoV‐2 Omicron variant infection.

	Normal range	Severe (*n* = 9)	Non‐severe (*n* = 16)	*p* value^a^
Procalcitonin, ng/mL	< 0.05	0.304 (0.13–0.77)	0.07 (0.18–0.327)	0.105
C‐reactive proteins, mg/L	0.00–4.00	25.52 (5.65–89.27)	3.94 (1.84–19.98)	0.091
WBC count, ×10^9^/L	4.00–10.0	8.78 ± 3.85	9.76 ± 4.97	0.589
Neutrophil granulocyte count, ×10^9^/L	2.00–7.00	6.44 ± 3.20	7.17 ± 3.68	0.611
Lymphocyte count, ×10^9^/L	0.80–4.00	1.12 ± 0.57	1.76 ± 0.99	0.053
Monocyte cell count, ×10^9^/L	0.12–1.0	0.55 (0.35–0.78)	0.65 (0.52–0.77)	0.350
Red blood cell, ×10^9^/L	4.00–5.00	4.22 ± 0.63	4.47 ± 0.70	0.377
Hemoglobin, g/L	120.00–160.00	122.56 ± 15.16	132.50 ± 21.39	0.191
Platelet count, ×10^9^/L	100.00–300.00	209.22 ± 101.70	265.69 ± 83.58	0.340
Alanine aminotransferase, U/L	5.00–40.00	33.60 (16.10–49.70)	15.95 (10.92–29.87)	0.126
Aspartate aminotransferase, U/L	8.00–40.00	44.90 (25.05–82.65)	17.20 (13.05–33.40)	0.037*
Creatine kinase, U/L	25.00–170.00	1186.20 (194.40–7281.75)	117.00 (54.40–542.17)	0.024*
Creatinine, μmol/L	21.00–68.00	71.00 (41.50–83.00)	75.00 (54.00–86.00)	0.333
Prothrombin time, s	10.60–14.30	13.70 (13.20–15.30)	12.85 (12.42–13.97)	0.113
Fibrinogen, g/L	2.00–4.00	4.33 ± 1.22	3.63 ± 1.53	0.221
Activated partial thromboplastin time, s	26.00–40.00	37.70 (32.00–38.85)	34.05 (32.07–37.75)	0.591
Thrombin time, s	14.00–21.00	16.30 ± 5.72	17.63 ± 1.65	0.386
D‐dimer, μg/mL	< 0.02	2.52 (0.52–7.96)	0.49 (0.24–0.95)	0.018*
Fibrin degradation products, μg/mL	0.00–5.00	5.56 (1.87–21.52)	2.00 (1.50–3.39)	0.169
Pulmonary infection on chest CT or radiography (*n* = 19)		7 (88)	8 (57)	0.193

^a^ *p < 0.05; **p < 0.01; ***p < 0.001.

*Note:* Data are represented as median (interquartile range), mean ± standard deviation, or *n* (%).

Abbreviations: CNS, central nervous system; CT, computed tomography; WBC, white blood cell.

### 3.3 CSF and Neurological Imaging Findings

**Figure 1:**
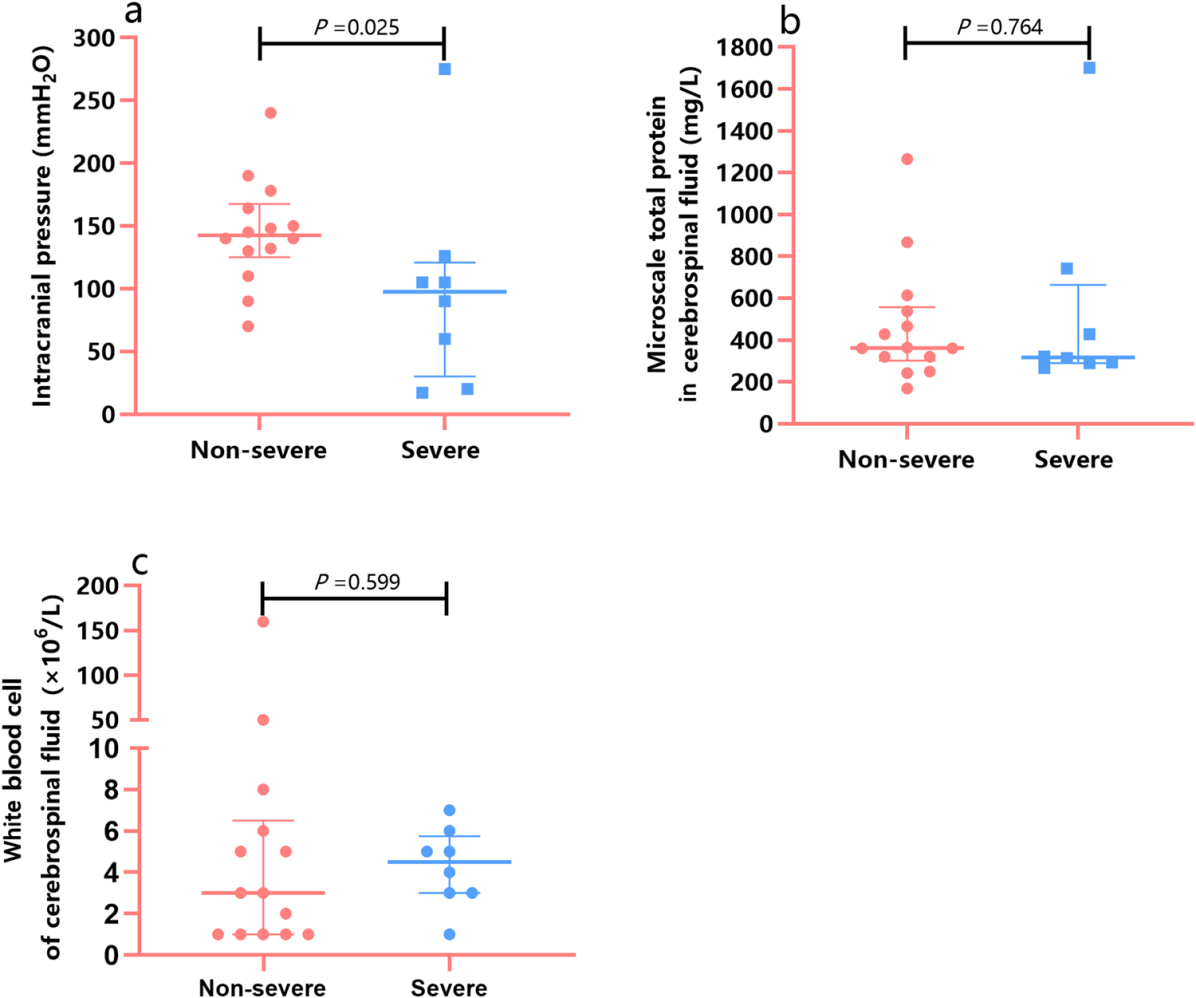
Cerebrospinal fluid test results in patients with SARS‐CoV‐2 Omicron infection‐related CNS complications. Of the 22 patients diagnosed with CNS complications who underwent lumbar puncture, 8 were classified as having severe CNS complications and 14 were classified as having nonsevere CNS complications. Intracranial hypertension (intracranial pressure > 180 mmH_2_O) occurred in 3/22 patients (14%), all of whom were in the nonsevere group. (a) Intracranial pressure: Overall, intracranial pressure was significantly higher in the nonsevere group than in the severe group. (b) CSF microscale total protein: Nine patients had elevated microscale total protein levels ( > 400 mg/L). (c) CSF white blood cell count: Two patients (9%) had elevated white blood cell counts ( > 8 × 10^6^/L). CNS, central nervous system; CSF, cerebrospinal fluid.

Of the 25 patients in the CNS complications group, 22 underwent lumbar puncture and CSF samples were collected for routine and biochemical examinations. Three (14%) patients had increased intracranial pressure ( > 180 mmH_2_O). Of the 22 patients who underwent lumbar puncture, 10 (45%) had abnormal CSF results; specifically, nine (41%) had elevated total protein levels ( > 400 mg/L), and two (9%) displayed elevated white blood cell counts in the CSF ( > 8 × 10^6^/L). Of the 22 patients with CNS complications who underwent lumbar puncture, 8 were classified as having severe CNS complications, and 14 were classified as having nonsevere CNS complications. Notably, no significant differences were observed between the two groups in CSF pressure, total protein levels, or white blood cell count (Table 4 and Figure 1). Furthermore, 17 patients underwent mNGS in the CSF, among whom two from the severe group had SARS‐CoV‐2 detected in the CSF, and three from the nonsevere group had autoantibodies indicative of autoimmune encephalitis (Table 4). Of the 25 patients in the CNS complications group, five exhibited extensive demyelination or white matter changes on neuroimaging, suggesting potential virus‐induced alterations.

**Table 4:** Symptoms and CSF findings in patients with CNS complications.

No.	Group	Intracranial pressure	CSF (*N* = 22)			Brain CT or MRI
			M‐TB	WBC	mNGS/Immunology	
1	Nonsevere	110	1265.6	50	N	N
2	Nonsevere	240	168.4	2	/	N
3	Severe	20	293.4	5	A^1^	A
4	Severe	90	428.7	6	/	N
5	Severe	275	313.5	5	/	A
6	Nonsevere	140	242.4	1	N	N
7	Nonsevere	148	319.7	1	N	N
8	Nonsevere	132	250.4	3	N	N
9	Nonsevere	90	361.1	3	N	N
10	Nonsevere	164	465.0	5	N	N
11	Nonsevere	178	428.8	1	N	N
12	Severe	126	1701.8	4	A^1^	A
13	Severe	105	742.4	7	/	N
14	Severe	17	289.4	3	N	N
15	Nonsevere	150	363.8	1	N	N
16	Severe	105	266.1	3	/	N
17	Nonsevere	190	867.4	160	A^2^	A
18	Severe	60	321.2	1	N	N
19	Nonsevere	70	614.2	5	A^2^	N
20	Nonsevere	145	360.8	8	A^2^	N
21	Nonsevere	130	537.3	6	N	A
22	Nonsevere	140	320.9	1	N	N

*Note:* The normal range of intracranial pressure is 80–180 mmH_2_O; the normal range of CSF micrototal protein (M‐TP) is 200–400 mg/L; and the normal range of WBC in the CSF is 0–8 × 10^6^/L.

Abbreviations: A, abnormal; A^1^, SARS‐CoV‐2 positive (Sequence Counts: No. 3 is 16, No.12 is 39); A^2^, autoimmune encephalitis antibody positive; CNS, central nervous system; CSF, cerebrospinal fluid; CT, computed tomography; M‐TP, microscale total protein; mNGS, metagenomic next‐generation sequencing; MRI, magnetic resonance imaging; N, normal; WBC, white blood cell.

## 4 Discussion

Age may influence the progression of COVID‐19‐related CNS complications. Previous studies have consistently identified advanced age as a significant risk factor for severe COVID‐19. Yet, limited evidence exists regarding its impact on the severity of neurological complications. In our study, older patients were more susceptible to severe CNS complications associated with the Omicron variant. A study by Sanchez et al. [15] demonstrated a significant correlation between impaired consciousness and advanced age. These findings collectively suggest that advanced age may be linked to the development of severe neurological symptoms in patients with SARS‐CoV‐2 Omicron variant infection. In elderly patients, neurons exhibit significantly reduced resilience to multiple stressors owing to impaired oxidative stress response, metabolic stress, and diminished cellular stress adaptation [16]. This neuronal vulnerability may heighten pathological susceptibility to SARS‐CoV‐2 infection. Furthermore, SARS‐CoV‐2 can invade the CNS through the blood‐brain barrier (BBB) [17]. These studies indicate that aging compromises the BBB integrity and induces BBB dysfunction, potentially exacerbating SARS‐CoV‐2 neuroinvasion and neurological complications in elderly patients with COVID‐19 infection [18] Consequently, older individuals have an increased risk of developing severe neurological disorders.

This study revealed that individuals diagnosed with COVID‐19 who also had two or more underlying medical conditions were more likely to develop severe COVID‐19‐related CNS complications. Liotta et al. [19] reported that the simultaneous presence of chronic underlying conditions, including hypertension, diabetes, and a medical history of neurological disorders, was a significant risk factor for developing COVID‐19‐related encephalopathy. Additionally, research has demonstrated that these conditions are more prevalent among patients with neurological symptoms associated with SARS‐CoV‐2 infection [20] Furthermore, patients with COVID‐19‐related neurological complications and a history of chronic underlying diseases have higher mortality rates [7] These findings indicate an association between chronic underlying diseases and the development and prognosis of COVID‐19‐related neurological complications, which are potentially linked to factors such as the body's pro‐inflammatory state, immune abnormalities, and endothelial dysfunction [21] Therefore, the implementation of early screening and monitoring protocols for patients with COVID‐19‐related CNS complications with concurrent chronic underlying diseases may positively affect illness prognosis.

No significant association exists between completion of booster immunization and CNS complication occurrence. As highlighted in a systematic review [22] the Omicron variant exhibits potent immune evasion capabilities and enhanced receptor binding affinity, which may substantially compromise vaccine efficacy. Andrews et al. [23] reported that third‐dose booster vaccination effectively enhances protection from infection. However, this effect wanes over time, which helps explain our findings. Nevertheless, vaccination remains clinically significant in reducing severe disease incidence and improving patient prognosis [24] Although our study demonstrated no significant difference in vaccination rates between severe and nonsevere CNS complications subgroups, this null finding might be attributed to an insufficiently large sample size.

A notable disparity in CK levels was observed between the COVID‐19 CNS complications and non‐CNS complications cohorts, suggesting a potential association with seizures in patients with CNS complications. Seizures represent the most frequently observed CNS manifestation. Most patients manifest focal or generalized epileptic seizures as the primary clinical presentation, despite the absence of a history of epilepsy. Omicron may increase the risk of seizures. Seizures among individuals with COVID‐19 may be attributable to excessive neuronal excitation triggered by a SARS‐CoV‐2 infection‐induced cytokine storm, wherein pro‐inflammatory cytokines (e.g., IL‐1β, IL‐6) enhance glutamate‐mediated excitability and impair GABAergic inhibition, collectively lowering seizure thresholds [25] A few case reports have documented that patients with COVID‐19‐associated CNS complications may present with seizures before the onset of altered consciousness [26, 27] Furthermore, new‐onset seizures could indicate severe systemic complications such as hypoxic encephalopathy, cerebrovascular events, and cytokine storm, potentially signaling a higher risk for adverse neurological outcomes in COVID‐19 [28] CK concentrations were significantly elevated in the severe CNS complications subgroup than in the nonsevere CNS complications subgroup. However, in patients with CNS complications, no significant difference was observed in the incidence of seizures between the severe and nonsevere groups. Furthermore, in addition to the increase in CK levels attributed to skeletal muscle injury resulting from seizures, a subset of patients with COVID‐19 may also develop rhabdomyolysis [29] subsequently leading to an increase in serum CK concentrations. Our study indicates that patients with COVID‐19‐related severe CNS complications may be at risk of developing rhabdomyolysis. However, further investigations with larger sample sizes are warranted to explore the relationship between rhabdomyolysis and COVID‐19 CNS complications.

In an investigation conducted by Tutal et al., [30] patients with COVID‐19‐related neurological complications had elevated serum d‐dimer levels within the subgroup affected by a cytokine storm, suggesting that elevated d‐dimer levels might be associated with the occurrence of a cytokine storm within the body. CNS complications are intrinsically linked to cytokine storms. Cytokine storms induced by SARS‐CoV‐2 infection have the potential to cause endothelial injury and alter microvascular permeability [31] Consequently, systemic coagulation dysfunction and widespread intravascular coagulation may occur, ultimately leading to multiorgan failure, with a higher incidence in critically ill individuals [32] In this study, d‐dimer levels were significantly elevated in the subgroup with severe CNS complications than in the nonsevere disease subgroup, indicating the presence of a hypercoagulable state and secondary fibrinolysis in patients with severe CNS complications following Omicron infection. The results of a retrospective study [10] conducted in Shanghai, China, revealed that not administering anticoagulant medication increased the risk of neurological complications, suggesting that early anticoagulant treatment may be beneficial for reducing the occurrence of neurological complications. However, research on the relationship between the severity of CNS complications following Omicron infection and hypercoagulable states is limited. Further investigations are needed to determine whether early administration of anticoagulant medications is beneficial in patients with severe COVID‐19‐related CNS complications.

The mechanisms by which Omicron induces CNS complications have not yet been definitively established. The prevailing theory posits that Omicron directly enters the CNS through nasal olfactory epithelial cells [17] However, in this study, only two patients with CNS complications had detectable levels of SARS‐CoV‐2 in their CSF, indicating that SARS‐CoV‐2 does not necessarily cause symptoms through direct invasion of the CNS. Khan et al. [33] analyzed the CSF from frontal lobe dissections performed in 35 patients with COVID‐19 and confirmed the absence of SARS‐CoV‐2 in most cases, as in our study. Another hypothesis is that COVID‐19 disrupts the BBB by causing a cytokine storm [34] This study revealed significantly elevated neutrophil and leukocyte counts in the CNS complications group. These increased peripheral inflammatory marker levels suggest that patients with CNS complications experience an inflammatory assault. Previous studies have shown a close association between CNS lesions and BBB disruption [35, 36] The body's inflammatory response can exacerbate the release of pro‐inflammatory factors, which can further attack and compromise the integrity of the BBB. In this study, CSF analysis of selected patients with CNS complications revealed abnormalities in cell counts and albumin levels, indicating a correlation between systemic inflammation and BBB disruption. Rhally et al. [37] identified an association between changes in the microstructure of the white matter in the anterior corona radiata and the genu of the corpus callosum and CRP levels in patients with COVID‐19‐related encephalopathy. This discovery provides evidence supporting the existence of a relationship between the occurrence of COVID‐19‐related CNS complications and the body's inflammatory response. Furthermore, SARS‐CoV‐2 is linked to autoimmune disorders of the CNS. In our study, autoimmune antibodies were identified in the CSF of three patients, likely mediated by a systemic hyper‐inflammatory response triggered by SARS‐CoV‐2 infection. This response may induce BBB dysfunction and intrathecal inflammation, thereby contributing to the pathogenesis of CNS autoimmunity [38, 39]

Lumbar puncture is an essential procedure for assessing intracranial lesions because it can reveal the underlying pathological and physiological changes within the cranium. Most of the 22 CSF specimens included in this study had normal white blood cell counts. This suggests that COVID‐19 encephalopathy does not significantly alter the number of white blood cells in the CSF, which is in accordance with the findings of Toklu et al. [40] However, in this study, approximately half of the patients had elevated CSF total protein levels. Similarly, CSF analysis of two patients with COVID‐19‐related CNS complications, reported by Andriuta et al., [41] also revealed elevated CSF total protein levels accompanied by normal white blood cell counts. This evidence suggests that SARS‐CoV‐2 can disrupt the BBB, with alterations in the CSF total protein being more commonly observed. We found no association between the results of conventional CSF analysis and the severity of CNS complications. A study of 150 CSF samples by Jarius et al. [42] revealed that the degree of neurological involvement was independent of the levels of intrathecal inflammation markers. Similarly, Espindola et al. [43] found that changes in the CSF among patients with COVID‐19 CNS complications were diverse and inconsistent. These results indicate that CSF analysis lacks specificity; therefore, routine CSF examinations do not reflect the severity of COVID‐19 CNS complications.

The intracranial pressure of cases with severe CNS complications appeared to be significantly lower than that of the nonsevere group. There may be two reasons for this. First, the overall statistical results may have been affected by the low intracranial pressure in two cases of severe CNS complications (cases number 3 and 14), which may have been caused by excessive dehydration before lumbar puncture or improper technical operation by the physicians. Second, severe cases of CNS complications tend to be dominated by cerebral edema rather than meningitis; therefore, intracranial pressure is often not significantly elevated. In fact, markedly elevated intracranial pressure is uncommon in patients with both severe and nonsevere CNS complications.

This study has two main limitations: a relatively small sample size and a retrospective nature. Thus, larger prospective studies are needed to address the limitations of this study.

## 5 Conclusions

Adults with CNS complications associated with Omicron variant infection often have concurrent COVID‐19 pneumonia, presenting with common symptoms such as fever, dizziness, and headache, which cannot be distinguished from the symptoms observed in patients with COVID‐19 without CNS complications. CNS complications in patients with Omicron infection may include seizures, syncope, impaired consciousness, and elevated blood PCT and serum CK levels. Severe CNS complications related to Omicron infection are more likely to occur in older individuals and in those with chronic underlying diseases. Serum d‐dimer, CK, and aspartate aminotransferase levels may be elevated in patients with severe CNS complications. Regardless of the severity, most adults with CNS complications have normal intracranial pressure and CSF white cell count, with elevated CSF total protein levels. In addition, the findings of routine CSF examinations do not reflect the severity of COVID‐19 CNS complications.

## Author Contributions

**Tingting Li:** investigation, writing – original draft. **Zhirong Deng:** investigation, writing – review and editing. **Qinfu Zhang:** data curation, writing – review and editing. **Xiaoying Qi:** writing – review and editing. **Wei Deng:** writing – review and editing. **Zunge Wu:** writing – review and editing. **Chuli Xiao:** writing – review and editing. **Weiqiang Zheng:** supervision, writing – review and editing. **Chuanghong Ke:** supervision, writing – review and editing. **Huanqin Han:** funding acquisition, project administration, supervision, writing – review and editing.

## Ethics Statement

The study was approved by the Ethics Committee of Guangdong Medical University (Approval No.: PJKT2024‐033) and was carried out in accordance with the guidelines of the Declaration of Helsinki.

## Conflicts of Interest

The authors declare no conflicts of interest.

## Supporting information

STROBE for Observational studies2.

## Data Availability

All data included in this study are available upon request by contact with the corresponding author.
